# Characterization of Poorly Cohesive and Signet Ring Cell Carcinomas and Identification of PTPRM as a Diagnostic Marker

**DOI:** 10.3390/cancers14102502

**Published:** 2022-05-19

**Authors:** Go Eun Bae, Sun Hyung Kang, Ju Seok Kim, Seok-Hwan Kim, Kyung-Hee Kim, Jin-Man Kim, Kwang-Sun Suh, Hyung Kyu Park, Dong-Wook Kang, Hyunjung Lee, Min-Kyung Yeo

**Affiliations:** 1 Department of Pathology, Chungnam National University School of Medicine, Munwha-ro 266, Daejeon 35015, Korea; goeunbae1@gmail.com (G.E.B.); phone330@cnu.ac.kr (K.-H.K.); jinmank@cnu.ac.kr (J.-M.K.); kssuh@cnu.ac.kr (K.-S.S.); elecmelon@gmail.com (H.K.P.); 2 Departments of Internal Medicine, Chungnam National University School of Medicine, Daejeon 35015, Korea; porrtos@daum.net (S.H.K.); showsik@hanmail.net (J.S.K.); 3 Department of Surgery, Chungnam National University School of Medicine, Daejeon 35015, Korea; kjxh7@naver.com; 4 Department of Pathology, Chungnam National University Sejong Hospital, 20 Bodeum 7-Ro, Sejong 30099, Korea; astro966@gmail.com; 5 Department of Pathology, Chungnam National University Hospital, Munwha-ro 282, Daejeon 35015, Korea; leehyunjung21@gmail.com

**Keywords:** gastric cancer, poorly cohesive, signet ring cell, transcriptome, diagnostic marker

## Abstract

**Simple Summary:**

The classification of signet ring cell (SRC) carcinoma has been inconsistent and SRC carcinoma was classified as a subtype gastric cancer of poorly cohesive (PC) carcinoma. SRC and PC gastric carcinomas are morphologically similar but has been suggested to exhibit different biological behavior. We compared clinical and molecular characteristics of SRC and PC carcinomas. SRC and PC carcinomas showed significantly different clinical behavior that SRC carcinoma was associated with favorable clinical factors, suggesting that these subtypes should be classified and treated differently. SRC and PC carcinomas shared common transcriptome expression patterns, however, PC carcinomas showed an increased expression of genes related to cancer progression. Among genes differentially expressed between PC and SRC carcinomas, protein tyrosine phosphatase receptor type M (PTPRM) was overexpressed in PC and related to unfavorable clinical factors. PTPRM was identified as a potential diagnostic and prognostic biomarker for PC carcinoma.

**Abstract:**

Background and aims. Signet ring cell (SRC) and poorly cohesive (PC) gastric carcinomas are morphologically similar but exhibit different biological behavior. We compared the clinical and molecular characteristics of SRC and PC carcinomas. Methods. Diffuse-type gastric cancer (GC) cases were classified into SRC carcinomas (>90% of SRCs), PC carcinomas (<10% of SRCs), and combined PC/SRC carcinomas (≤90% but ≥10% of SRCs). The gene expression patterns in SRC and PC carcinomas were examined by transcriptome and protein immunohistochemistry analyses, and diagnostic and prognostic biomarkers were identified. Results. SRC and PC carcinomas showed significantly different clinical behaviors but shared common RNA expression patterns. PC carcinomas showed an increased expression of genes related to cancer progression. Among genes differentially expressed between PC and SRC carcinomas, protein tyrosine phosphatase receptor type M (PTPRM) was overexpressed in PC and related to unfavorable clinical factors. Conclusion. We found that PC and SRC carcinomas had distinct clinical characteristics and should be classified as different carcinoma types. PTPRM was identified as a potential diagnostic and prognostic biomarker for PC carcinomas and could represent a potential therapeutic target.

## 1. Introduction

Gastric cancer (GC) is the fifth most common cancer and the fourth leading cause of cancer-related death globally [1]. Although the incidence of GC has decreased in the last 50 years, the incidence of diffuse-type GC continues to increase [2]. Thus, understanding the characteristics of diffuse-type GC is essential for improving the prognosis and treatment of this disease. Lauren et al. [3] classified signet ring cell (SRC) and poorly cohesive (PC) carcinomas as subtypes of diffuse-type GC. SRC carcinoma is named for its resemblance to a signet ring because of the large amount of mucin in the cytoplasm that displaces the nucleus to the cell periphery, whereas PC carcinoma is characterized by diffuse distribution of tumor cells, which lack cellular cohesion and are isolated from each other. 

SRC carcinoma is classified as a subtype of PC carcinoma in the fourth edition of the World Health Organization (WHO) classification [4]. However, SRC carcinoma was re-classified as a distinct subtype of GC in the fifth edition, as in the previous third WHO edition [5,6]. In the past few decades, the classification of SRC carcinoma has been inconsistent. SRC carcinoma has been categorized as PC, undifferentiated or poorly differentiated (PD) carcinoma, possessing variable proportions of other types of PD carcinoma components. Controversies exist not only in the terminology, but also in the biological behavior and prognosis of SRC and PC carcinomas. The prevalence of SRC carcinoma of the stomach ranges from 3.4% to 39% [7]. The prognostic relevance of SRC is also unclear, and the poor prognostic impact of SRC GC remains controversial [7,8,9].

Recently, gastric SRC, and PC carcinomas have attracted increasing interest. The International Gastric Cancer Association European Chapter (IGCA) presented a consensus on the pathological definition of SRC, PC, and combined PC and SRC (combined PC/SRC) carcinomas for the standardization of terminology and classification [10]. In addition, an extended indication for endoscopic surgical resection of SRC carcinoma was proposed based on several endoscopic studies [11]. SRC carcinomas show distinct mutational patterns associated with specific clinicopathological characteristics that differ from those of PC carcinomas [12].

There are limited data on the differences between SRC and PC carcinomas, which, although morphologically similar, exhibit different biologic behaviors. Here, we compared the clinical and molecular features of SRC with those of PC and combined PC/SRC carcinomas. A comprehensive pathologic review of SRC, PC, and combined PC/SRC carcinomas was performed to elucidate the distinct behavior and characteristics of SRC and PC carcinomas. To analyze the genetic differences between SRC and PC carcinomas, we performed transcriptome analysis and identified potential diagnostic and prognostic biomarkers for gastric SRC and PC carcinomas according to mRNA expression and protein immunohistochemistry (IHC).

## 2. Materials and Methods

### 2.1. GC Cases

All GC cases that underwent endoscopic resection or gastrectomy between June 2019 and July 2021 at Chungnam National University Hospital (Daejeon, Korea) were reviewed. A total of 1029 GC cases were enrolled, and hematoxylin eosin-stained slides were reviewed by two experienced gastrointestinal pathologists (Go Eun Bae and Min-Kyung Yeo) and re-classified according to the IGCA classification [10] as follows: SRC carcinoma (>90% of SRCs), PC carcinoma (<10% of SRCs), and combined PC/SRC carcinoma (≤90% but ≥10% of SRCs). The clinicopathologic characteristics of GC cases were collected from medical records. The GC stage was determined according to the American Joint Committee on Cancer TNM criteria in the cancer staging system, eighth edition [13]. This retrospective study was approved by the Chungnam National University Hospital Institutional Review Board (IRB file no. CNUH 2019-11-043), which waived the requirement for informed consent. All samples were provided by the Biobank of Chungnam National University Hospital, a member of the Korea Biobank Network.

### 2.2. Analysis of the mRNA Expression of Genes Related to Cancer Progression Using the NanoString Assay

Among 1029 cases, 24 pairs of cancer and normal gastric tissue samples that were stored in our institution as formalin-fixed paraffin-embedded (FFPE) blocks were used for transcriptome analysis. The selected 24 cases were stage 1a to rule out influencing factors and they had enough tissue samples for RNA extraction. Six cases of SRC carcinoma without lymph node metastasis, 12 cases of PC carcinoma (six cases with lymph node metastasis and six without lymph node metastasis), and six cases of combined PC/SRC carcinoma were selected. All 24 cases of GC carcinomas were stage T1a (tumor confined to the mucosa); of these, 18 cases had no lymph node metastasis (N0), and six cases of PC carcinoma were N1 (one lymph node metastasis) stage. Whole FFPE tissue blocks were sectioned on coated slides, and GC and normal areas were micro-dissected from the slides. RNA was extracted using the Qiagen RNeasy Kit (Qiagen) according to the manufacturer’s instructions and subjected to hybridization. Transcriptome analysis was performed using the nCounter pancancer progression panel kit (Nanostring Technology Inc., Seattle, WA), which includes 770 genes (Table S1). For hybridization, 5 µL of each RNA sample was mixed with 8 µL of nCounter reporter probes in hybridization buffer and 2 µL of nCounter capture probes (for a total reaction volume of 15 µL) for 18 h at 65 °C. Excess probe was removed using two-step magnetic bead-based purification on the Prep Station. The abundance of specific target molecules was quantified on the nCounter Digital Analyzer by counting the individual fluorescent barcodes and assessing the target molecules. For each assay, a high-density scan was performed encompassing 280 fields of view. Data were collected using the nCounter Digital Analyzer after obtaining images of the immobilized fluorescent reporters in the sample cartridge with a CCD camera. mRNA data analysis was performed using nSolver (version 4.0) software, which is available from NanoString Technologies. The mRNA profiling data were normalized using housekeeping genes. Paired cancer and normal tissue samples from each case were compared, and RNA expression patterns were compared between SRC, PC, and combined SRC/PC carcinoma subgroups.

### 2.3. Quantitative Real-Time Reverse-Transcription Polymerase Chain Reaction (qRT-PCR) 

Twenty-four cases evaluated by the NanoString assay were subjected to quantitative real-time reverse-transcription polymerase chain reaction (qRT-PCR) analysis of target genes using endoscopic biopsy samples, which were performed prior to surgery. Total RNA was extracted from GC cancer samples using a Qiagen kit (Valencia, CA, USA) and reverse transcribed to cDNA using the Superscript^TM^ II RT-PCR System (Invitrogen, Karlsruhe, Germany) according to the manufacturer’s recommendations. Real-time quantitative PCR was performed using the ABI PRISM 7900HT Sequence Detection System (Applied Biosystems, Foster City, CA, USA). Optimum reaction conditions were obtained using 5 µL of Universal Master Mix (Applied Biosystems) containing dNUTPs, MgCl2, reaction buffer, AmpliTaq Gold, 90 nM of primer(s), and 250 nM fluorescence-labeled TaqMan probe. Table S2 shows the results of TaqMan real-time PCR assay. The results were normalized against GAPDH gene expression (endogenous control). The △Ct value was determined by subtracting the △Ct of the control sample from the individual △Ct of test samples. The relative quantification values of target genes were compared, and significant values were defined as fold change ≥2 or ≥3 and fold change ≤0.5.

### 2.4. Immunohistochemical Staining

Of the 1029 cases, a total 47 cases of SRC and PC carcinomas were selected for the immunohistochemical study. The selected 47 cases (12 SRC carcinomas and 35 PC carcinomas cases) had enough tissue samples for immunohistochemical study. The samples consisted of 12 SRC carcinomas and 35 PC carcinomas. Whole FFPE tissue blocks were sectioned on coated slides, deparaffinized with xylene, and hydrated using serial solutions of alcohol. The sections were heated in a pressure cooker (containing 10 mmol/L sodium citrate, pH 6.0) for 3 min for antigen retrieval and incubated in 0.03% hydrogen peroxide for 10 min to block endogenous peroxidases. The sections were incubated at room temperature for 1h with the following primary antibodies (Table S3): aminopeptidase (ANPEP), prokinetin (PROK2), calcineurin B homologous protein isoform 2 (CHP2), protein tyrosine phosphatase receptor type M (PTPRM), amphiregulin (AREG), and COL7A1. Liver hepatocytes and tonsil tissues in the FFPE samples were used as the positive and negative controls, as recommended in the antibody datasheets and test validation results using human tissue samples in the laboratory setting. The staining intensities of ANPEP, PROK2, CHP2, PTPRM, AREG, and COL7A1 were designated as negative, weak, moderate, or strong and the area stained was quantified in increments of 5% across a 0–100% range. A histoscore (H-score), a summation of the proportion of the area stained at each intensity level multiplied by the weighted staining intensity (e.g., 0, negative; 1, weak; 2, moderate; and 3, strong), was generated [14]. For categorical analyses, immunohistochemical expression at the median value was based on high or low PTPRM and PROK2 expression for comparative analysis. The Human Protein Atlas (https://www.proteinatlas.org/ENSG00000173482-PTPRM/tissue, accessed on 6 April 2022), an interactive web tool that assesses human tissue proteins, was used to determine the expression level of proteins in GC.

### 2.5. Statistical Analysis

The nSolver analysis software performed a two-tailed t-test on the log-transformed normalized data assuming unequal variance. Associations between protein levels and the clinicopathological parameters of GC were examined using Spearman rank correlation coefficients, Mann–Whitney *U*-tests, and Kruskal–Wallis tests. The Wilcoxon signed rank test was used for group comparisons. All analyses were performed using SPSS 24.0 (SPSS Inc., Chicago, IL, USA).

## 3. Results

### 3.1. Clinical Characteristics of SRC, PC, and Combined PC/SRC Carcinomas

Among the 1029 GC cases enrolled, 543 (52.8%) underwent endoscopic dissection and 486 (47.2%) underwent surgical tumor removal. The clinicopathological characteristics of the GC subtypes are summarized in Table 1. GCs were diagnosed as tubular adenocarcinoma (TAC) (79%), combined PC/SRC carcinoma (6.9%), mixed carcinoma (combined TAC and PC or SRC carcinoma components) (4.1%), PC carcinoma (3.8%), Epstein Barr virus-associated carcinoma (3.4%), SRC carcinoma (1.6%), and others including mucinous and undifferentiated carcinomas (1.3%). The incidence of mixed carcinoma was 4.1% and comprised TAC with PC or SRC carcinoma components, of which components consisted of PC (43%), SRC (31%), and combined PC/SRC (26%) components.

**Figure 1:**
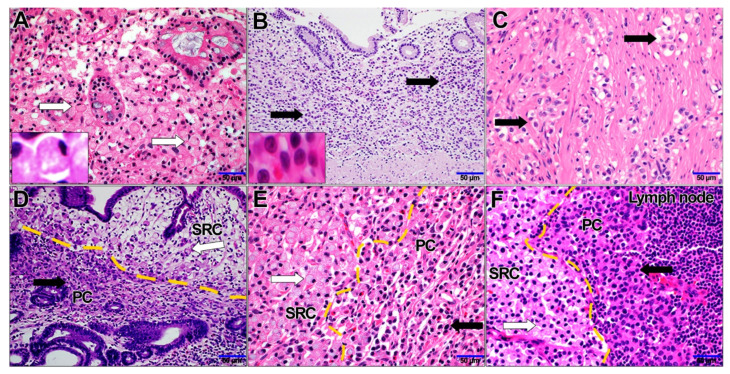
Representative images of (**A**) signet ring cell (SRC) carcinoma (white arrow) and (**B**) poorly cohesive (PC) carcinoma (black arrow) of the mucosa. (**C**) PC carcinomas infiltrating the muscularis propria. Combined PC and SRC carcinoma located (**D**) in the mucosa, (**E**) admixed in the invasive front, and (**F**) in the lymph node metastasis (**A**–**F**) Hematoxylin eosin-stained slides, ×400).

The clinical characteristics of patients with SRC, PC, and combined PC/SRC carcinomas are summarized in Table 2. A total of 126 cases of SRC, PC, and combined PC/SRC carcinomas were enrolled and the incidence rates of SRC, PC, and combined PC/SRC carcinomas were 1.6%, 3.8%, and 6.9% of all GC cases, respectively. All GC patients were Asian and from Korea. Patient age ranged from 36 to 85 years, with a mean age of 60 years, and the male/female ratio was 2.1:1. Endoscopic resection was performed in 27 patients (21.49%), and 99 patients (78.6%) underwent surgical removal. SRC carcinomas were significantly associated with early GC (*p* = 0.000), negative lymph node metastasis (*p* = 0.001), lower T stage, and lower pathologic stage (*p* = 0.004) compared with PC and combined PC/SRC carcinomas. All SRC carcinoma cases were confined to the mucosa (T1a) without lymph node metastasis (Figure 1A). PC carcinoma was associated with the increased invasiveness of GC, including a greater depth of invasion (*p* = 0.001), lymph node metastasis (*p* = 0.001), and advanced pathologic stage (*p* = 0.004) compared with SRC carcinoma and combined PC/SCR (Table 2, Figure 1B,C). The clinicopathological characteristics of combined PC/SRC carcinoma included those of PC and SRC carcinomas (Table 1). Combined PC/SRC showed two infiltration patterns as follows: (i) SRC confined to the mucosa with PC infiltrated to the invasive front (Figure 1D) or (ii) admixed SRC and PC components (Figure 1E). The nodal metastatic tumors of combined PC/SRC carcinoma showed the two following patterns: (i) nodal metastatic tumors showing the primary PC carcinoma (cases such as “i” in the above description) or (ii) nodal metastatic tumors consisting of both SRC and PC components of the primary tumor pattern (cases such as “ii” in the above description) (Figure 1F). The proportion of SRC components (10–90%) in the combined PC/SRC carcinomas had no effect on lymph node metastasis or advanced stage (*p* = 0.543 and *p* = 0.642).

### 3.2. Transcriptome Analysis of SRC and PC Carcinomas

**Figure 2:**
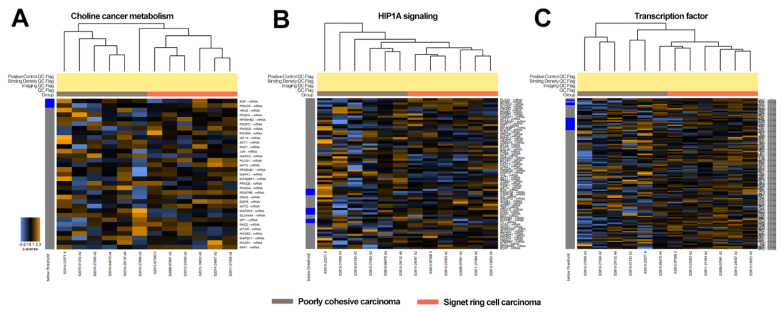
Expression of cancer progression-related genes in signet ring cell carcinoma and poorly cohesive carcinoma. Genes related to (**A**) choline cancer metabolism, (**B**) HIP1A signaling, and (**C**) transcription factor.

To better characterize the differences between SRC, PC, and combined PC/SRC carcinomas, gene expression profiles related to cancer progression were investigated using 24 pairs of tumor and normal gastric tissue samples. The 770 genes and signaling pathways related to cancer progression are listed in Table 1. The gene expression signatures of the SRC, PC, and combined PC/SRC carcinomas shared similar transcription profiles and were under the same branch (Figures S1 and S2). However, PC carcinomas were slightly enriched in genes related to choline cancer metabolism, HIP1A signaling, and transcription factors (Figure 2). Genes related to choline cancer metabolism (Figure 2A (from top to bottom): *EGF, PRKCG, HRAS, PDGFA, RPS6KB2, PDGFC, PIK3CD, PIK3R5, HIF1A, AKT1, RAC1, JUN, MAPK3, PLCG1, AKT3, RPS6KB1, MAPK1, EIF4EBP1, PRKCB, PIK3CA, PDFGRB, KRAS, EGFR, AKT2, MAP2K2, SLC44A4M SP1, RAC2, MTOR, PIK3R2, MAP2K1, PIK3R1, RAF1),* HIP1A signaling (Figure 2B: *PLCG1, EP300, PIK3CG, PLCG2, PIK3R2, MTOR, ERBB2, PIK3R1, MAP2K1, AKT3, RPS6KB1, MAPK1, IGF1, EIF4EBP1, EGLN3, TLR4, PRKCB, ENO2, PIK3CA, VEGFA, CDKN1A, AKT2, EGFR, PDK1, CYBB, HK2, MAP2K2, EIF4E2, PGK1, STAT3, LDHA, ALDOA, CAMK2D, MAPK3, VHL, AKT, RBX1, TCEB1, ENO1, TIMP1, HIF1A, EGF, CAMK2A, CAMK2B, IL6, ANGPT2, NFKB1, TCEB2, NOS3, TEK, PRKCG, FNG, ENO3, HK3, PFKFB4, PFKFB1, TF, NOS2, HKCD1, SERPINE1, SLC2A1, PIK3CD, ANGPT1, PIK3R5, HMOX1, CREBBP, FLT1, RPS6KB2, EDN1*), and transcription factor (Figure 2C: *SOX17, CD34, WNT5A, WWTR1, AEBP1, NOTCH1, GREM1, ZEB1, RUNX1, SRF, CD44, SMAD5, SANI1, CTNNB1, ETV4, TWIST2, BMP7, DD42, NAA15, STA3, SOX17, SNAI3, TCF4, ZEB2, PROM1, MYC, IRF6, EPAS1, MMP14, SOX2, RBL2, RORA, HIP1A, ICAM1, MTA1, HOXB3, NR4A3, SMAD9, SANI2, PKNOX1, ID2, ELK3, TP53, MEOX2, ENO1, TWIST1, RUX1T1, MED1, RBL1, SOX9, HOXA5, GRHL2, RBPJ, SP1, ID4, NFKB1, SMAD3, ID1, GATA4, RORB, NR3C1, PLA2G10, NFATC2, GTF2I, SMAD3, MAPK1, ADD1, SMAD4, SMAD1, MAF, HDAC4, KDM1A, MTOR, TFDP1, PKN1, ELF3, SPDEF, PTTG1, CHD4, TCF3, MED23, NR4A1, ISL1, NFAT5, FLI1, EP300, TAL1, BRMS1, CD36, JUN, STAT1, PYCARD, FOXO4, CREBBP, MTDH, PRF1, VHL, DNPP2, RB1*) were listed. The gene expression pattern was similar between combined PC/SRC and PC carcinoma (Figure S2). No significant differences in gene expression were detected between PC carcinoma with and without lymph node metastasis (Figure S3).

**Figure 3:**
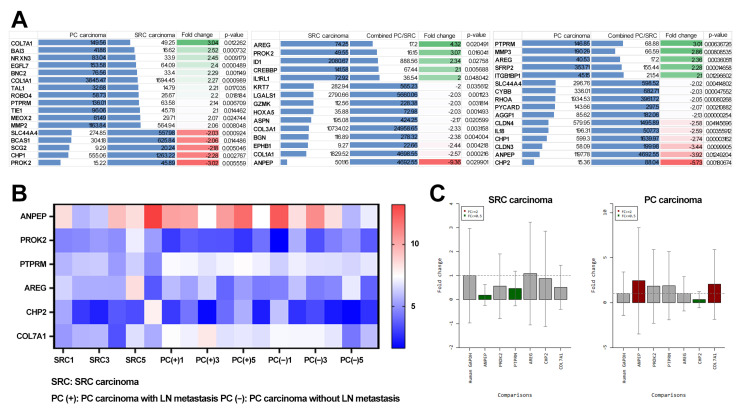
(**A**) Gene expression patterns of signet ring cell (SRC) carcinoma, poorly cohesive (PC) carcinoma, and combined PC and SRC carcinoma using Nanostring assay. (**B**,**C**) *ANPEP, PROK2, PTPRM, AREG, CHP2,* and *COL7A1* gene expression in SRC and PC carcinomas was compared using (**B**) Nanostring and (**C**) RT-PCR assay.

To identify genes specific to each histological subtype, we generated a list of upregulated or downregulated genes according to a threshold fold change of ≥2 between SRC and PC carcinomas (Figure 3A). *COL7A1, BAI3, NRXN3, EGFL7, BNC3, COL1A1, TAL1, ROBO4, PTPRM, TIE1, MEOX2*, and *MMP2* were upregulated in PC carcinoma, whereas *PROK2, CHP1, SCG2, BCAS1,* and *SLC44A4* were upregulated in SRC. Comparison of PC and SRC carcinomas with combined PC/SRC carcinoma showed that *PTPRM, MMP3, AREG, SFRP2*, and *ITGB1BP1* were upregulated in PC carcinoma, whereas *AREG, PROK2, ID1, CRDBBP*, and *IL1RL1* were upregulated in SRC carcinoma.

Next, we applied stringent criteria with a threshold fold change of ≥3 and selected six upregulated or downregulated transcripts (Figure 3B). Genes encoding *ANPEP, PROK2, CHP2, PTPRM, AREG,* and *COL7A1* showed significant differential expression in SRC and PC carcinomas. *PTPRM, AREG,* and *COL7A1* were upregulated in PC carcinoma compared with SRC carcinoma. Conversely, expression of genes encoding for *PROK2, CHP1,* and *AREG* was downregulated in PC carcinoma compared with SRC carcinoma.

### 3.3. Identification of Diagnostic Markers for SRC and PC Carcinoma

We showed that the genes encoding *ANPEP, PROK2, CHP2, PTPRM, AREG,* and *COL7A1* were differentially expressed between SRC and PC carcinomas (Figure 3A). To determine whether these genes could be used as diagnostic markers to decide on the optimum treatment, we assessed their expression using endoscopic biopsy samples using qRT-PCR (Figure 3C). In endoscopic biopsy samples, the expression of genes encoding *ANPEP* and *COL7A1* was higher in PC carcinoma than in SRC carcinoma when the threshold was set at a fold change of ≥2. The expression of genes encoding *CHP2* was lower in PC carcinoma than in SRC carcinoma, whereas the expression of genes encoding *ANPEP* and *PTPRM* was lower in SRC carcinomas than in PC carcinoma when the threshold was set at a fold change of ≥2.

### 3.4. Immunohistochemical Analysis of SRC and PC Carcinomas and its Clinical Significance

**Figure 4:**
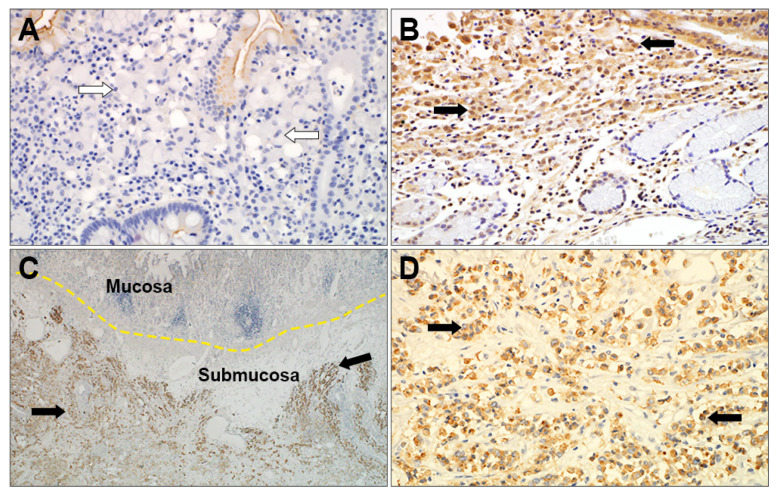
Representative images of PTPRM immunohistochemical staining. PTPRM showed (**A**) negative staining of the signet ring cell carcinoma (white arrow) and (**B**) positive staining of the poorly cohesive carcinoma (black arrow) in the mucosa. PTPRM highlighted the infiltrating pattern of (**C**) poorly cohesive carcinomas in the submucosa (×200) and (**D**) poorly cohesive carcinoma in the invasive front (×400).

Immunostaining for ANPEP, PROK2, CHP2, PTPRM, AREG, and COL7A1 was performed in 47 cases of SRC and PC carcinomas. ANPEP, PROK2, CHP2, PTPRM, and AREG showed a cytoplasmic and membranous staining pattern (Figure 4), whereas COL7A1 staining was not detected in cancer cells and it was thus excluded from further evaluation. PROK2, CHP2, and PTPRM immunohistochemical expression was higher in PC than in SRC (*p* = 0.018, 0.010, and 0.018, respectively; Table S4). CHP2 immunohistochemical staining was stronger in PC carcinoma, which was not consistent with the gene expression assay results. CHP2 was thus excluded as a candidate diagnostic marker.

Analysis of clinicopathological features relative to PTPRM and PROK2 immunohistochemical expression (Table 3) showed that PTPRM was positively correlated with the incidence of PC carcinoma and lymph node metastasis (*p* = 0.001 and *p* = 0.005, respectively) and showed a slight relation to advanced GC (*p* = 0.059). PROK2 expression was positively correlated with the incidence of PC carcinoma (*p* = 0.015), but not correlated with lymph node metastasis or an advanced stage (*p* = 0.210 and *p* = 0.709, respectively).

The protein expression patterns of the selected genes in GC were validated using The Human Protein Atlas (Figure S4). PTPRM was expressed in PD carcinoma (CAB022442, Patient id: 2105) and PC carcinoma (CAB022442, Patient id: 2326) (Figure S4A,B), and high PTPRM expression levels were related to poor overall survival in GC (Figure S4C).

## 4. Discussion

GC was classified into two major subgroups “intestinal/diffuse” and “differentiated/undifferentiated” carcinomas according to the formation of tubular or papillary structures [3,15]. The recent fifth edition of the WHO classification includes several GC histologic variants based on the predominant histologic patterns of GC carcinomas [16]. The WHO classification defines SRC carcinoma as the presence of at least 50% signet ring cells in the pathologic specimen, and SRC carcinoma could contain <50% TAC or PC components. Studies assessing the prognosis of patients with SRC carcinoma have reported conflicting results [7,8,9]. Recently, SRC carcinoma with >90% of SRC components was classified as “pure SRC carcinoma” and shown to have a significantly lower incidence of lymph node metastasis and better survival than PC carcinoma and combined PC/SRC carcinoma related to the proportion of the SRC [17,18]. To provide insight into the clinical behavior of SRC carcinoma, we reviewed SRC and PC carcinomas after standardization of the histological definition.

In this study, SRC carcinoma was more common among younger patients and was associated with favorable clinical factors; it showed an intra-mucosal tumor location without lymph node metastasis. The pattern of invasion of SRC carcinoma consisted of horizontal spread without vertical invasion. By contrast, PC carcinoma was associated with unfavorable clinical factors, a greater depth of invasion, frequent lymph node metastasis, and an advanced pathologic stage. The survival rate was significantly higher for patients with SRC carcinoma than for patients with PC or combined PC/SRC carcinoma [17]. SRC and PC carcinomas showed significantly different clinical behavior, suggesting that these subtypes should be classified and treated differently.

SRC and PC carcinomas showed common morphologic features, including a lack of cellular cohesion. In this study, combined PC/SRC carcinoma (6.9%) was more common than PC or SRC carcinomas (1.6% and 3.8%). Combined PC/SRC showed two invasive patterns as follows: (i) SRC carcinoma confined to the top of the mucosal layer and PC carcinoma in the invasive front or (ii) both components were intermingled and showed deeper invasion. Our review of histologic slides of GC showed that advance stage GC cases with SRC carcinoma components always had PC carcinoma components. These data suggested that in combined PC/SRC, the SRC morphology was lost or trans-differentiated into PC morphology during tumor growth.

SRC and PC carcinomas showed similar gene expression patterns, which resulted in the inability to distinguish SRC, PC, and combined PC/SRC based on differential gene expression. However, PC carcinomas showed a slightly increased expression of genes related to cancer progression, such as genes involved in choline cancer metabolism, HIP1A signaling, and transcription factors. A targeted sequencing comparison showed that SRC and PC carcinoma shared a common mutational signature including *TP53, APC, KIT, EGFR*, and *PIK3CA*, and the frequency of mutations was higher in PC carcinoma than in SRC carcinoma [12]. In this study, the clinical behavior and transcriptome expression pattern of combined PC/SRC carcinoma was between those of PC and SRC carcinomas. This suggested that SRC carcinoma is closely related to PC carcinoma, and SRC might acquire invasive properties during clonal evolution or trans (or dual) differentiation to PC carcinoma associated with the upregulation of cancer progression-related genes.

In this study, the incidence rate of SRC and PC carcinomas was 1.6% and 3.8% of GCs, which was lower than that reported previously. The incidence of SRC carcinoma is 26% in the United States, 37% in Europe [8,19], 9.1% in China, and 8.3% in Japan [20,21]. Thus, ethnicity should be considered as a factor affecting the incidence of SRC carcinoma [19]. Combined PC/SRC carcinoma also showed a lower incidence (6.9%) in this study. The inconsistent prognostic impact of SRC carcinoma in previous studies, which showed a favorable prognosis in the early stage and adverse prognosis in advanced stages, could be related to the underestimation of combined PC/SRC carcinoma in the advanced stages [10]. The incidence of SRC was inversely related to an advanced tumor stage [17], and mixed carcinoma showed an aggressive behavior and frequent lymph node metastasis, as well as an advanced pathologic stage [4,22]. Mixed carcinoma accompanying SRC may have been classified as SRC in previous studies, which could affect the poor clinical impact of SRC carcinoma.

Because SRC and PC carcinomas differed significantly in clinical behavior, we analyzed genes that were upregulated or downregulated in PC and SRC to identify potential diagnostic markers. PTPRM was overexpressed and immunologically readily evaluable for protein expression in PC carcinoma compared with SRC carcinoma, both in surgical and endoscopic biopsy samples. PTPRM expression was related to the incidence of PC carcinoma and related to unfavorable clinical factors and poor overall survival, suggesting its potential as a diagnostic and prognostic biomarker for gastric SRC and PC carcinomas.

PTPRM is a member of the protein tyrosine phosphatase (PTP) family, a large family of enzymes; it is involved in cell–cell adhesion in epithelial and cancer cells and regulates cell growth, differentiation, and oncogenic transformation [23,24]. PTPRM is a tumor-associated factor, and its role in cancer has been investigated in several malignancies. PTPRM overexpression is negatively correlated with the progression of colon, neuroendocrine, and breast cancers [25,26,27]. However, PTPRM is positively correlated with poor prognosis in cervical cancer [28]. In this study, PTPRM expression was increased in GC, especially in PC carcinoma, and related to unfavorable prognosis. The role and precise mechanism of PTPRM in cancer remain unknown, and further exploration is necessary to elucidate its role in SRC and PC carcinomas.

The present results have clinical implications regarding the design of treatment strategies for different types of GC based on endoscopic biopsy and surgical samples. SRC carcinoma diagnosed using endoscopic biopsy can be considered an extended indication for endoscopic submucosal dissection [18], whereas a diagnosis of PC carcinoma should be an indication for surgical treatment. PTPRM expression could be an adjunctive marker for the diagnosis of PC carcinoma using biopsy and surgical samples as well as for predicting the prognosis of GC. A recent clinical trial assessing the response to chemotherapy showed a dependence on the type of cancer especially for SRC carcinoma [29]. Therefore, GC with different proportions of SRC or PC carcinoma components may show different responses to chemotherapy, and SRC and PC carcinoma classification is important for the design of therapeutic strategies.

This study had several limitations. The morphologic similarity of PC and SRC carcinomas could be confusing even with a clear definition of PC, SRC, and combined PC/SRC carcinoma. Inter- or intra-observer variability could thus limit the consistent diagnosis of GC. As endoscopic biopsy samples did not represent the whole GC tissue sample, pathologic diagnosis based on endoscopic biopsy could be limited and may have complicated decision making regarding treatment. The lack of GC cell lines specific for SRC or PC carcinomas made it difficult to evaluate the mechanism of target markers.

## 5. Conclusions

This study was the first to evaluate RNA expression specific to SRC and PC carcinomas. PC and SRC carcinomas showed distinct clinical characteristics, suggesting that they should be classified into different subtypes in order to allow appropriate decisions to be made about surgical treatment and predict patient prognosis. Further studies are necessary to identify additional genes affecting lymph node or distant metastasis in PC carcinoma as well as to elucidate the role and precise mechanism of PTPRM in GC. The present findings provided insight into the clinical and molecular signatures of SRC and PC carcinomas. Additionally, the present study identified a diagnostic and prognostic biomarker for PC carcinoma, PTPRM, that could potentially be a target for the development of novel cancer therapeutics.

## Figures and Tables

**Table 1:** Clinicopathologic characteristics of gastric cancer patients (*n* = 1029).

Characteristics	Gastric Cancer Patients								
	TAC	SRC Carcinoma	PC Carcinoma	Combined PC/SRC Carcinoma	Mixed Carcinoma	EBV-Associated Carcinoma	Others	Total	*p*
Sex									0.000
Male	626 (77.0)	8 (50.0)	18 (46.2)	38 (53.5)	25 (59.5)	31 (88.6)	12 (92.3)	758 (73.7)	
Female	187 (23.0)	8 (50.0)	21 (53.8)	33 (46.5)	17 (40.5)	4 (11.4)	1 (7.7)	271 (26.3)	
Age									0.000
≤61	355 (43.7)	13 (81.3)	23 (59.0)	49 (69.0)	30 (71.4)	26 (74.3)	6 (76.2)	502 (48.8)	
>61	458 (56.3)	3 (18.8)	16 (41.0)	22 (31.0)	12 (28.6)	9 (25.7)	7 (53.8)	527 (51.2)	
EGV vs. AGC									0.000
EGC	723 (88.9)	16 (100)	17 (43.6)	51 (71.8)	32 (76.2)	21 (60.0)	4 (30.8)	864 (84.0)	
AGC	90 (11.1)	0 (0)	22 (56.4)	20 (28.2)	10 (23.8)	14 (40.0)	9 (69.2)	165 (16.0)	
T stage									0.000
T1	724 (89.1)	16 (100)	17 (43.6)	51 (71.8)	32 (76.2)	21 (60.0)	4 (30.8)	865 (84.1)	
T2	45 (5.5)	0 (0)	1 (2.6)	4 (5.6)	7 (16.7)	4 (11.4)	0 (0)	61 (5.9)	
T3	19 (2.3)	0 (0)	8 (20.5)	8 (11.3)	1 (2.4)	8 (22.9)	5 (38.5)	49 (4.8)	
T4	25 (3.1)	0 (0)	13 (33.3)	8 (11.3)	2 (4.8)	2 (5.7)	4 (30.8)	54 (5.2)	
LN metastasis									0.000
Absent	742 (91.3)	16 (100)	20 (51.3)	54 (76.1)	30 (71.4)	27 (77.1)	4 (30.8)	893 (86.8)	
Present	71 (8.7)	0 (0)	19 (48.7)	17 (23.9)	12 (28.6)	8 (22.9)	9 (69.2)	136 (13.2)	
Pathologic stage									0.000
I–II	774 (95.2)	16 (100)	25 (64.1)	60 (84.5)	38 (90.5)	32 (91.4)	7 (53.8)	952 (92.5)	
III–IV	39 (4.8)	0 (0)	14 (35.9)	11 (15.5)	4 (9.5)	3 (8.6)	6 (46.2)	77 (7.5)	

TAC, tubular adenocarcinoma; SRC, signet ring cell; PC, poorly cohesive; mixed carcinoma, combined TAC and PC or SRC carcinoma; others, others including mucinous carcinoma and undifferentiated carcinoma; EGC, early gastric cancer; AGC, advanced gastric cancer; LN, lymph node.

**Table 2:** Clinicopathologic characteristics of SRC, PC, and combined PC/SRC gastric carcinomas (*n* = 126).

Characteristics	Gastric Cancer Patients				
	SRC Carcinoma	PC Carcinoma	Combined PC/SRC Carcinoma	Total	*p*
Sex					0.759
Male	8 (50.0)	18 (46.2)	38 (53.5)	64 (50.8)	
Female	8 (50.0)	21 (53.8)	33 (46.5)	62 (49.2)	
Age					0.254
<61	13 (81.3)	23 (59.0)	46 (69.0)	85 (67.5)	
≥61	3 (18.8)	16 (41.0)	22 (31.0)	41 (32.5)	
Surgical treatment					0.012
Endoscopic resection	8 (50)	7 (17.9)	12 (16.9)	27 (21.4)	
Gastrectomy	8 (50)	32 (82.1)	59 (83.1)	99 (78.6)	
EGV vs. AGC					0.000
EGC	16 (100)	17 (43.6)	51 (71.8)	84 (66.7)	
AGC	0 (0)	22 (56.4)	20 (28.2)	42 (33.3)	
T stage					0.001
T1	16 (100)	17 (43.6)	51 (71.8)	84 (66.7)	
T2	0 (0)	1 (2.6)	4 (5.6)	5 (4.0)	
T3	0 (0)	8 (20.5)	8 (11.3)	16 (12.7)	
T4	0 (0)	13 (33.3)	8 (11.3)	21 (16.7)	
LN metastasis					0.001
Absent	16 (100)	20 (51.3)	54 (76.1)	90 (71.4)	
Present	0 (0)	19 (48.7)	17 (23.9)	36 (28.6)	
Pathologic stage					0.004
I–II	16 (100)	25 (64.1)	60 (84.5)	101 (80.2)	
III–IV	0 (0)	14 (35.9)	11 (15.5)	25 (19.8)	

SRC, signet ring cell; PC, poorly cohesive; EGC, early gastric cancer; AGC, advanced gastric cancer; LN, lymph node.

**Table 3:** Correlation between PTPRM and PROK2 immunohistochemical expression and clinicopathologic factors in SRC and PC gastric carcinomas (*n* = 47).

Characteristics	Patients		PTPRM	Patients		PROK2		
	No. (%)	Low	High	*P*	No. (%)	Low	High	*P*
GC subtype				0.001				0.015
PC carcinoma	35 (74.5)	13 (54.2)	22 (95.7)		35 (74.5)	15 (60.0)	20 (90.9)	
SRC carcinoma	12 (25.5)	11 (45.8)	1 (4.3)		12 (25.5)	10 (40.0)	2 (9.1)	
EGV vs. AGC				0.059				0.319
EGC	25 (53.2)	16 (66.7)	9 (39.1)		25 (53.2)	15 (60.0)	10 (45.5)	
AGC	22 (46.8)	8 (33.3)	14 (60.9)		22 (46.8)	10 (40.0)	12 (54.5)	
T stage				0.192				0.706
T1–T2	27 (57.4)	16 (66.7)	11 (47.8)		27 (57.4)	15 (60.0)	12 (54.5)	
T3–T4	20 (42.6)	8 (33.3)	12 (52.2)		20 (42.6)	10 (40.0)	10 (45.5)	
LN metastasis				0.005				0.210
Absent	28 (59.6)	19 (79.2)	9 (39.1)		28 (59.6)	17 (68.0)	11 (50.0)	
Present	19 (40.4)	5 (20.8)	14 (60.9)		19 (40.4)	8 (32.0)	11 (50.0)	
Distant metastasis				0.276				0.237
Absent	43 (91.5)	23 (95.8)	20 (87.0)		43 (91.5)	24 (96.0)	19 (86.4)	
Present	4 (8.5)	1 (4.2)	3 (13.0)		4 (8.5)	1 (4.0)	3 (13.6)	

PC, poorly cohesive; SRC, signet ring cell; EGC, early gastric cancer; AGC, advanced gastric cancer; LN, lymph node.

## Data Availability

The raw data were generated at CNUH and derived data supporting the findings of this study are available from the corresponding author on request.

## Supplementary Materials

The following supporting information can be downloaded at: https://www.mdpi.com/article/10.3390/cancers14102502/s1, Figure S1: Expression comparison of genes in SRC carcinoma and PC carcinoma with/without lymph node metastasis, Figure S2: Expression comparison of genes between SRC carcinoma, PC carcinoma, and combined PC/SRC carcinoma, Figure S3: Expression comparison of genes between PC carcinoma with/without lymph node metastasis, Figure S4: PTPRM expression in GC from the Human Protein Atlas (A) PTPRM was expressed in PD carcinoma (CAB022442, Patient id: 2105), (B) in PC carcinoma (C) AB022442, Patient id: 2326), and (C) high PTPRM expression levels were related to poor overall survival in GC, Table S1: Lists of 770 genes of pancancer progression panel, Table S2: TaqMan real-time PCR assay probes, Table S3: Antibodies for IHC, Table S4: Pearson correlations of IHC results.
