# Supplementary material for: Characterization of Poorly Cohesive and Signet Ring Cell Carcinomas and Identification of PTPRM as a Diagnostic Marker
Source: Cancers (Basel). 2022 May 19;14(10):2502. doi: 10.3390/cancers14102502 (PMC9139305; doi:10.3390/cancers14102502)
Supplement: Supplementary file 1 [file cancers-14-02502-s001.zip › cancers-1695937-supplementary figures_R.pdf]

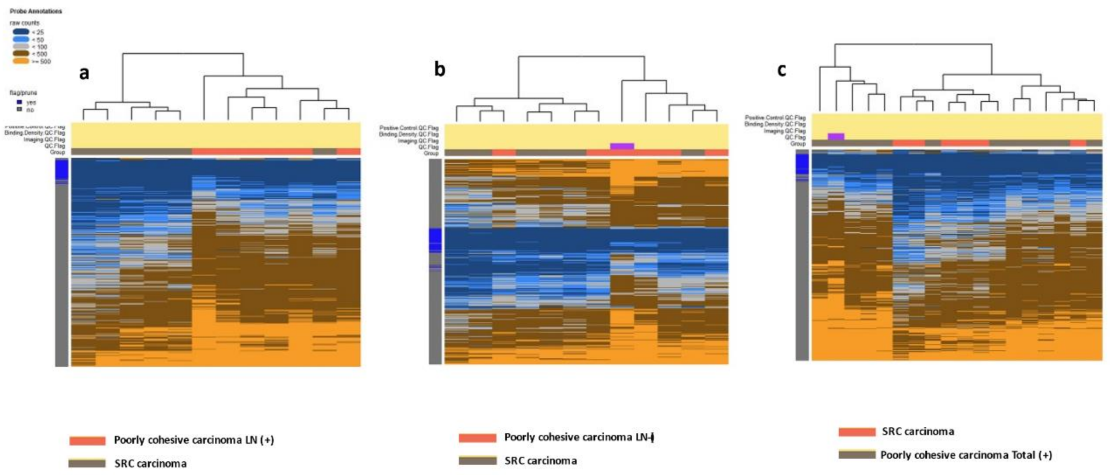

Figure S1. : Expression comparison of genes in SRC carcinoma and PC carcinoma with/without lymph node metastasis.

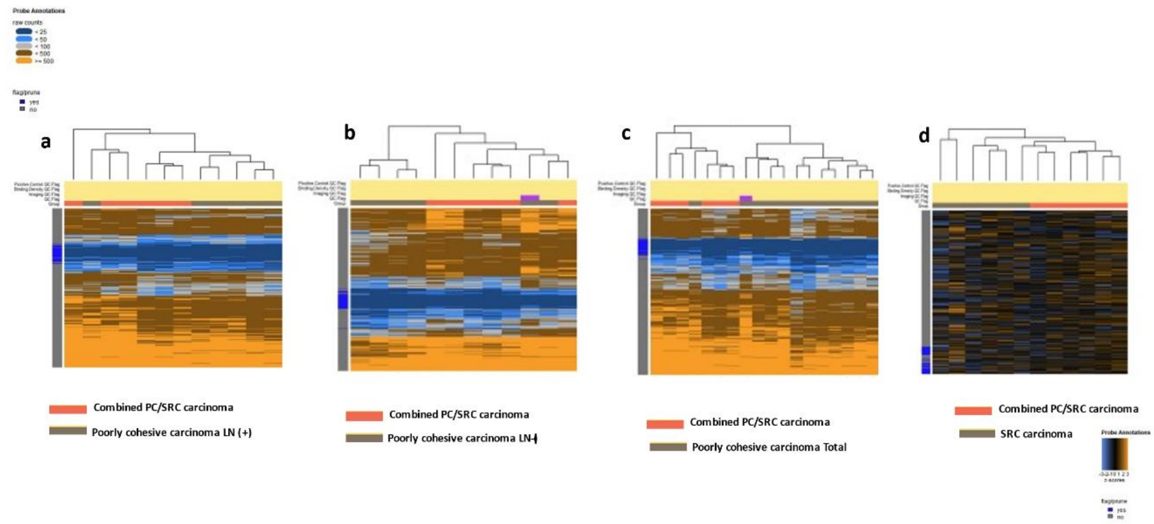

Figure S2. Expression comparison of genes between SRC carcinoma, PC carcinoma, and combined PC/SRC carcinoma.

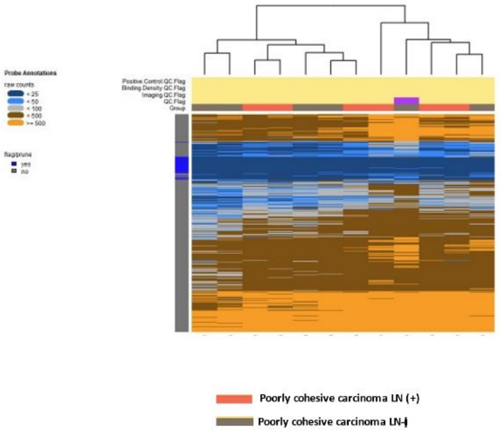

Figure S3. Expression comparison of genes between PC carcinoma with/without lymph node metastasis..

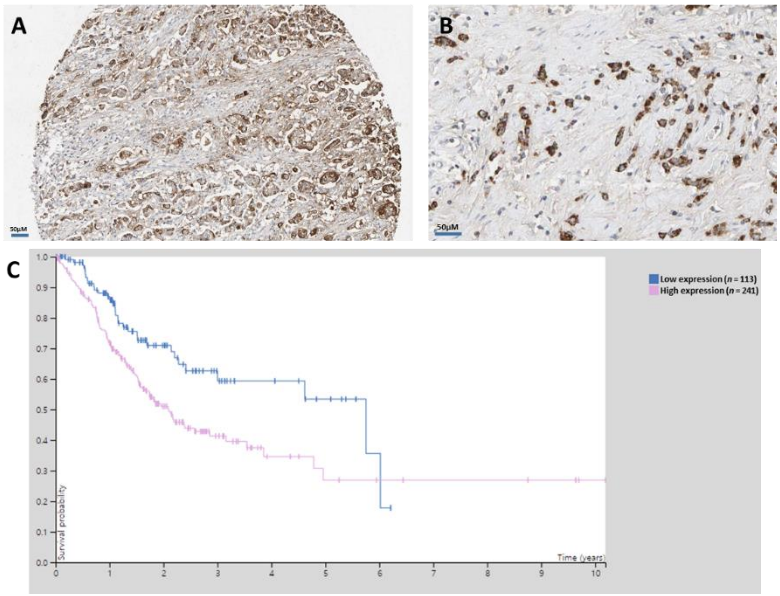

Figure S4. PTPRM expression in GC from the Human Protein Atlas (**A**) PTPRM was expressed in PD carcinoma (CAB022442, Patient id: 2105), (**B**) in PC carcinoma (CAB022442, Patient id: 2326), and (**C**) high PTPRM expression levels were related to poor overall survival in GC
